# Effect of angiotensin converting enzyme inhibitor on glomerular hyperfiltration in patients with type 1 diabetes

**DOI:** 10.12669/pjms.323.9399

**Published:** 2016

**Authors:** S. A. Jaffar Naqvi, Shahid Ahsan, Asher Fawwad, Abdul Basit, A Samad Shera

**Affiliations:** 1 S. A. Jaffar Naqvi, Chief Executive, The Kidney Foundation, Karachi, Pakistan; 2 Shahid Ahsan, MBBS, MPhil (Biochemistry), MPhil (NCD). Associate Professor, Dept. of Biochemistry, Hamdard College of Medicine and Dentistry, Hamdard University, Karachi, Pakistan; 3 Asher Fawwad, PhD. Associate Professor, Baqai Medical University, Senior Research Scientist, Research Department, Baqai Institute of Diabetology and Endocrinology, Karachi, Pakistan; 4 Abdul Basit, FRCP. Professor of Medicine, Department of Medicine Baqai Institute of Diabetology and Endocrinology, Baqai Medical University, Karachi, Pakistan; 5 A Samad Shera, FRCP. Honorary President (IDF), Secretary General (DAP), Director WHO Collaborating Centre, Diabetic Association of Pakistan, 5-E / 3, Nazimabad, Karachi-74600, Pakistan

**Keywords:** Angiotensin converting enzyme inhibitor, Glomerular hyperfiltration, Type 1 diabetes

## Abstract

**Objective:**

To assess the effect of angiotensin converting enzyme inhibition on glomerular filtration rate (GFR) in normotensive patient with type 1 diabetes.

**Methods:**

A two year non-placebo control prospective study was conducted after ethical approval at Diabetes Centre of Diabetic Association of Pakistan, a WHO collaborating centre in Karachi, Pakistan. All patients with type 1 diabetes visited the out-patients department from August 2009 till July 2011 and those who fulfilled the inclusion criteria were invited to participate. A total of 121 people aged ≥18 years and ≥ 5 years of diabetes were included. Pregnant and lactating woman and those aged <18 years were excluded. GFR was calculated by using CKD-EPI formula (eGFR) at baseline and after two year. On the basis of estimated GFR, patients at baseline were divided according to KDIGO classification of chronic kidney diseases into, hyperfiltration (eGFR ≥ 100 ml/min) and normal filtration group (eGFR < 100 ml/min). All subjects in hyperfiltration group received ACE inhibitor (treatment group) while patients with normal filtration did not receive ACE inhibitor (control group).

**Results:**

Fifty two patients (43%) were in the treatment and sixty nine (57%) were in the control group. At baseline eGFR, systolic and diastolic blood pressures between groups were non-significantly different. After two years, compared to baseline, eGFR of the treatment group declined and the control group increased significantly. No significant difference in systolic while diastolic blood pressure of the treatment group increased significantly after two years compared to baseline. In contrast both systolic and diastolic blood pressure of control group increased significantly after two years compared to their baseline values.

**Conclusion:**

Present study demonstrated that initiation of ACEI in hyperfiltration stage declined GFR and keep blood pressure within normal range.

## INTRODUCTION

Prevalence of diabetes is increasing worldwide.^1^ An estimate in 2013 illustrated 382 million people globally had diabetes with the projection of 592 million people by the year 2035. In Pakistan nearly 6.7 million people has diabetes that is expected to rise to 12.8 million by the year 2035. Among total estimated population of diabetes, nearly 497,000 have type 1 diabetes. With an annual incidence rate of 3%, nearly 79,000 new cases of type 1 diabetes to the pool per annum.^2^ Of the total estimated population of children with type 1 diabetes, 23 % are from the South East Asia Region.^2^ These children with type 1 diabetes are prone to develop diabetes related complications that pose burden on the individual, health care system and on the society. It is currently estimated that between 25-40% of patient with type 1 diabetes developed diabetes kidney disease.^3^ With this incidence and given that nearly 497 thousand individuals have type 1 diabetes worldwide, the prevention of diabetic kidney disease represent an over whelming clinical priority.

Diabetic nephropathy, a micovascular complication of diabetes, causes progressive decline in renal function (chronic kidney disease) that culminate to end stage renal disease (ESRD).^4^ Diabetes is one of the leading causes of renal disease worldwide contributing nearly 30-40% of all cases.^5^ Among patients who developed diabetic kidney diseases nearly 0.8% patients developed end stage renal disease (ESRD) needing dialysis or renal replacement treatment. Both are expensive treatment modalities.^6^

Pakistan is a developing country where average per capita yearly income is $1094.^7^ Nearly 36% of the population earns< 105 US$ per annum and more than 50% of the population lives below poverty line.^7^ A person on renal failure in Pakistan annually spent U$3000 only for dialysis.^8^ The cost of kidney transplantation and post transplantation medicine nearly cost about US$ 5000 and $ 2000 annually respectively.^9^ The low per capita income and high cost of renal failure treatment and poorly developed under resourced health care infrastructure make the problem more complex. Thus RRT if given to all ESRD patients; it required nearly half of our health budget of 60 billion for the management of this progressive disease only. And even after kidney transplantation, 5 years post transplantation mortality rate is double in diabetics compared to non-diabetics even in the developed country.^10^

An alternate approach to lessen this burden of disease is tertiary prevention of diabetes.^11^ Recent studies demonstrated that the onset and course of diabetic nephropathy can be ameliorated to a very significant degree by several interventions.^12^ These interventions would have their greatest impact if instituted at a point very early in the course of development of this complication. There is clear evidence for the pathogenic role of renin angiotensin system (RAS) in the development of DN.^13^ These data also provide a strong rationale for early and sustained blockade of the RAS for the primary preservation of kidney diseases in all patients with diabetes.^14^ However, clinical trial evidence to support this goal is inconsistent and limited. Furthermore its interpretation has been made more difficult because RAS blocker also lower BP, a key pathogenic factor in itself that contribute to the development of micro-albuminuria in diabetic patients. Indeed it has been argued that the better effect of RAS blockade on BP may partly explain the so called independent benefit with respect to the primary prevention of kidney disease.^15^

Nephropathy in patients with type 1 diabetes as suggested by Mogensen progress through various stages of declining GFR except the first stage called glomerular hyperfiltration.^16^ Glomerular hyperfiltration is a well-established phenomenon occurring early in some patients with type 1 diabetes. A meta-analysis documented that rate of GFR decline is greater in patients with hyperfiltration.^17^ Hence, present study was designed to find out the frequency of patients with type 1 diabetes in hyperfiltration state and to demonstrate the effect of ACE inhibitor in the preservation of GFR and blood pressure over two years of follow up period.

## METHODS

This was a two year non-placebo control prospective study approved ethically by Institutional Review Board of Diabetic Association of Pakistan (DAP) – World Health Organization (WHO) Collaborating Centre in Karachi, Pakistan. A cohort of type 1 diabetic patients attending the outpatient clinic of DAP was assembled in August 2009 and followed till July 2011. After excluding the pregnant and lactating type 1 diabetic woman and those with less than 18 years of age, a total of one hundred and twenty one (n=121) type 1 diabetic subjects with ≥ 5 years history of diabetes and ≥ 18 years of age were included in the study. Screening for nephropathy was done by assessing serum creatinine and estimating GFR at baseline and after two year. GFR was calculated by using CKD-EPI formula.

On the basis of estimated GFR, patients at baseline were divided according to KDIGO classification of chronic kidney diseases^18^,^19^ into, hyperfiltration (eGFR ≥ 100 ml/minutes) and normal filtration group (eGFR < 100 ml/minutes). All subjects in hyperfiltration group received ACE inhibitor while patients in normal filtration group did not receive ACE inhibitor and served as a control group.

All data entered and analyzed by SPSS v 16.0 and presented as Mean±SD or n (%) where appropriate. Comparison between hyperfiltration and normal filtration groups at baseline and comparisons of eGFR, systolic and diastolic blood pressure of both groups with their respective baseline values were done by application of student-t test. A p-value of <0.05 was considered statistically significant.

## RESULTS

Baseline characteristics of the study participants are shown in Table-I. A total of 121 subjects were included in the study. Among them nearly 43% (n=52) patients had eGFR value ≥100 ml/minutes while 57% (n=69) had below 100 ml/minutes. At baseline except eGFR no significant difference were found in mean age, duration of diabetes, SBP and DBP, fasting and random blood glucose and serum creatinine level. Compared to baseline, two years of treatment with ACEI to hyperfiltration group brought significant decline in mean GFR. While, mean GFR of the patients in control arm (without ACEI) increased significantly. Systolic blood pressure of the patient in the hyperfiltration group showed non-significant difference after two years of ACEI treatment while diastolic blood pressure is significantly increased compared to baseline. On the contrary both systolic and diastolic blood pressure increased significantly in patients without ACEI after two years compared to their baseline value.

**Table-I:** Baseline characteristics of the study participants (n=121) (year: 2009).

*Variables*	*eGFR≥ 100ml/min n=52(42.9%)*	*eGFR < 100ml/min n=69(57.0%)*	*p-value*
Age (years)	24.60±5.94	26.97±13.31	0.233
Duration of Diabetes (years)	12.25±8.40	13.63±9.36	0.583
Gender			
Male	34	33	0.054
Female	18	36	
Weight (kg)	37.13±15.38	37.71±14.99	0.835
Systolic blood pressure (mmHg)	107.30±13.06	104.23±11.29	0.180
Diastolic blood pressure (mmHg)	72.32±8.65	73.54±9.42	0.478
Fasting blood glucose (mg/dl)	172.49±91.81	178.04±94.52	0.761
Random blood glucose (mg/dl)	192.26±82.78	203.34±99.26	0.529
Serum Creatinine (mg/dl)	0.85±0.09	0.99±0.41	0.049
eGFR	113.75±10.71	75.12±16.40	0.000

Data presented as Mean±SD.

P<0.05 considered as statistically significant.

Data presented as Mean±SD.

GFR; glomerular filtration rate, ACEI; Angiotensin converting enzyme inhibitor.

## DISCUSSION

In this prospective study nearly 43 % (n=52) of patients with type 1 diabetes were found to have GFR value ≥ 100 ml/minutes. Patients in the treatment arm showed significant decline in mean GFR value after two years of follow up compared to baseline value. In contrast GFR of the patients in control (normal filtration) group, increased significantly suggesting Reno-protective effect of ACE inhibitors in hyperfiltration stage. A number of studies elucidated the potential role of ACEI or ARBi as Reno-protective medicine.^20^ In these studies appearance of micro-albuminuria was used as a surrogate end point of the studies. In patients with type 1 diabetes an increase in albumin excretion has traditionally been taken as equivalent to decline in GFR. However the term micro-albuminuria, macro-albuminuria implies but does not confirm the onset of decline in GFR.^21^ Studies now suggest that appearance of micro-albuminuria per se may not be taken as an equivalent of declining GFR due to intermittent nature of micro-albuminuria. Later it was also found that nearly 50-60% of these micro-albuminuria patients revert back to normo-albuminuria spontaneously even without use of rennin-angiotensin system inhibitor.^21^ Like others this is also supported by a study conducted by Lervang et al., who failed to demonstrate an association between the development of increased urinary albumin excretion rate (AER) and glomerular hyperfiltration in an 18 year retrospective study on type 1 diabetic patients.^22^ Findings of present data is consistent with the concept that increased in AER can occur without parallel changes in GFR and vice versa and thus both should not be consider as mutually exclusive parameters.

**Table-II:** Comparison of eGFR and blood pressure at baseline and after two years.

	*2009*	*2011*	*p-value*
eGFR			
eGFR (≥100ml/min) With ACEI n=52	113.75±10.71	108.46±14.92	0.009
eGFR (<100ml/min) Without ACEI n=69	75.12±16.40	84.16±24.13	0.001
*Systolic Blood Pressure*			
eGFR (≥100ml/min) With ACEI n=52	107.21 ±12.71	109.95±11.67	0.080
eGFR (<100ml/min) Without ACEI n=69	104.96±14.07	111.02±15.03	<0.0001
*Diastolic Blood Pressure*			
eGFR (≥100ml/min) With ACEI n=52	73.82±9.44	76.56±7.88	0.011
eGFR (<100ml/min) Without ACEI n=69	74.17±10.17	77.00±8.35	0.002

Data presented as Mean±SD.

P<0.05 was considered as statistically significant.

GFR; glomerular filtration rate, ACEI; Angiotensin converting enzyme inhibitor

In the present study we therefore measured GFR at baseline and two years later after initiation of ACEI and found that mean GFR of hyperfiltration group after two years of treatment declined significantly as compared to the group without ACEI whose mean GFR after two years of follow up increased significantly. One may attribute this decline of GFR in hyperfiltration group to the better glycemic or blood pressure control. Nevertheless we assured blindness of both patients and treating physician about renal status of the patients. Moreover baseline characteristic of the two groups of patients showed non-significant difference in the mean fasting and random blood glucose and systolic and diastolic blood pressure, eliminating the potential source of bias that could be present in the study otherwise.

Studies found hyperfiltration has been more prevalent in those with recent onset of diabetes and when glycemic control is poor.^17^,^23^ It would therefore be essential to compare the glycemic status and duration of diabetes at entry into the study as patients with worst glycemic control and short duration of diabetes were more likely to have hyperfiltration. In the present study both glycemic status and duration of diabetes between two groups (normal filtration vs hyperfiltration groups) showed non-significant difference suggesting glycemic status per se is not the mediator of hyperfiltration. A finding supported by Ficociello et al., 2009 in a fifteen years follow up study on 426 type 1 patients selected from the First Joslin Kidney Study.^24^ He concluded that hyperglycemia predicted the onset of micro-albuminuria, but not hyperfiltration, so the effect of glycemic control is not through an effect on GFR. In contrast to tubular hypothesis, hemodynamic hypothesis of hyperfiltration in patients with type 1 diabetes suggested that hyperfiltration in patients with type 1 diabetes is due to changes in pre-glomerular (afferent tone) and post glomerular (efferent tone) vascular tone. This tone is regulated by vasoactive mediators. Change in the level of vasoactive mediators has been postulated to be mediated by hyperglycemia accounted for hyperfiltration in diabetes. Duration of diabetes thus influences the development of hyperfiltration by long exposure of unnoticed hyperglycemic period which accounted for prolong exposure to vasoactive mediators leading to hyperfiltration in patients with diabetes.^25^

ACEI is considered a drug of choice for the patients with diabetes and blood pressure. In the present study mean systolic and diastolic blood pressure of the patients of treatment group were in the normotensive range at baseline that fall after two years of treatment non-significantly, further strengthening the concept that Reno-protective effect of ACEI is not mediated only by regulating blood pressure.^26^

Studies have documented that hypertension (HTN) affects nearly 70% of the patients with diabetes that increases the risk of development of nephropathy.^27^ HTN occurs early and is a major determinant of the rate of nephropathy progression. Both cross sectional and longitudinal studies identified the correlation between HTN and decline in renal function in type 1 patients with greater reduction occurred in those who had hyperfiltration.^28^

In the present study both mean SBP and DBP of the patients in hyperfiltration group (treatment arm) showed non-significant difference compared to baseline value. In contrast both SBP and DBP of the control group rose significantly after two years of follow up. Though in the scientific community, question about initiation of ACEI to a diabetic patient is still under discussion. Some suggest ACEI should be started when patient develop micro- albuminuria, other support addition of ACEI when patient develop hypertension. Still others suggest initiation of ACEI even before appearance of micro-albuminuria or development of hypertension i.e. in hyperfiltration stage.^29^,^30^ Findings of the present study are in agreement with the growing convincing evidence that suggested that ACEI should be initiated to a diabetic patient before development of micro-albuminuria or hypertension in a stage when GFR is found beyond and above normal range.

### Strength and Limitation

Selection of patients with type 1 diabetes and prospective design are the main strength of our study. However our findings demands careful interpretation as certain biochemical test like HbA1c and micro-albuminuria was not performed at baseline and during follow up period. Moreover, as reference value of GFR of native population is not available we used arbitrarily defined values for characterization of normal and hyperfiltration group.

## CONCLUSION

Results of the present study has demonstrated that initiation of ACEI in hyperfiltration stage reduced GFR and keep blood pressure within normal range. Findings have thus supported that every type 1 diabetic patients should receive ACEI as an integral part of their therapy.
